# Arterial inflammation after myocardial infarction: regulating the immune system

**DOI:** 10.1038/s41392-026-02628-1

**Published:** 2026-03-24

**Authors:** Christos P. Kotanidis, Charalambos Antoniades

**Affiliations:** Division of Cardiovascular Medicine, Radcliffe Department of Medicine, Acute Multidisciplinary Imaging & Interventional Centre, University of Oxford, Oxford, UK

**Keywords:** Cardiology, Immunotherapy, Drug discovery, Translational research

In a recent study published in Nature Medicine by Sriranjan-Rothwell et al.^1^ selectively expanding regulatory T cells (*T*_reg_) with low-dose interleukin 2 (IL-2) led to a decrease in arterial inflammation measured on PET-CT imaging of the ascending aorta and common carotid arteries.

T_reg_ cells are a specialized FOXP3⁺ CD4⁺ T-cell lineage that enforces immune tolerance and restrains excessive inflammation, while effector T cells (*T*_eff_) are activated conventional T cells that execute pro-inflammatory and cytotoxic programs when engaged. IL-2 sits at the center of this axis (Fig. 1): at high doses it can activate *T*_eff_ cells, whereas *T*_reg_ cells can be preferentially expanded with carefully titrated low doses of IL-2.^2^ Consistent with this biology, prior clinical experience has shown that low-dose IL-2 can be administered safely and produces a selective, statistically significant increase in circulating *T*_reg_ cells.

In atherosclerosis, reduced *T*_reg_ cell number and impaired *T*_reg_ cell function have been associated with disease activity, and experimental data support a role for *T*_reg_ cells in dampening plaque inflammation and promoting features of stability through pathways that include IL-10, TGF-β and checkpoint signaling, such as CTLA-4.^2^ After a myocardial infarction (MI), T_reg_ cells can traffic to the injured myocardium and restrain excessive inflammation and fibrosis, in part by skewing macrophages toward reparative phenotypes and by limiting CD8⁺ T-cell mediated damage-effects that have translated into improved structural and functional outcomes in preclinical models.^2^ These mechanistic links make IL-2 particularly attractive as a therapeutic agent because low-dose regimens can tip the immune equilibrium toward regulation without broadly suppressing host defence.

The IVORY trial^1^ was a phase 2 randomized controlled trial that recruited patients presenting with unstable angina, NSTEMI, or STEMI (most treated with percutaneous coronary intervention)selected for residual inflammation defined by high-sensitivity C-reactive protein (hsCRP) of more than 2 mg/L. At total of 63 patients were included in the analysis and started dosing with either low-dose IL-2 (1.5 × 10^6^ IU subcutaneous injection) or placebo in the form of subcutaneously administered 5% glucose within 14 days of the index admission to hospital. An [^18^F]FDG PET-CT scan of the ascending aorta and carotids was performed before the start of treatment and at the end of an 8-week treatment period, that included once daily injections for 5 days during an induction phase, followed by once-weekly injections for 7 weeks during a maintenance phase.

A significant reduction in arterial inflammation from baseline to follow-up was observed in both groups, with this difference being greater in the low-dose IL-2 arm compared to placebo. At the end of 8 weeks of treatment, levels of arterial inflammation were lower in patients treated with low-dose IL-2 (mean TBR_max_:2.04 ± 0.28) compared to placebo (mean TBR_max_:2.22 ± 0.25). These differences were more pronounced in the specific vascular segment that was the most inflamed (i.e., had the highest TBR_max_ value) at baseline.

As expected, low-dose IL-2 led to a significant increase in circulating *T*_reg_ cells, while changes in other T cells subsets (including central memory, effector memory, Th2, and Th17) did not differ meaningfully between groups. T follicular helper (T_FH_) cells interestingly increased from baseline in the placebo arm whereas they decreased in the low-dose IL-2 arm, however the specific role and dynamics of T_FH_ cells following myocardial infarction remain incompletely characterized. Traditional clinical and laboratory measures, including circulating neutrophils, hsCRP, lipids, and left ventricular ejection fraction, did not differ significantly between groups at the end of treatment. Injection site reaction, bruising and fatigue were the most common adverse events. Infections were mild, with no difference observed in the frequency of infections between the two groups. Finally, 55 patients were followed up for 2 years for a composite endpoint of cardiovascular death, resuscitated cardiac arrest, nonfatal myocardial infarction, ischemic stroke or unplanned coronary revascularization, which occurred in 3 (11%) patients in the placebo group and none in the low-dose IL-2 arm.

Several limitations warrant consideration. The cohort was small and baseline inflammatory burden was high (median hsCRP at screening 9.55 mg/L with a median of 11 days from admission to first dose). Sex imbalance (predominantly male participants), limited ethnic diversity, and low prevalence of diabetes mellitus restrict generalizability. Notably, patients with insulin-treated diabetes were excluded because of methodological interference with PET imaging, which limits the applicability of these findings to a real-world acute coronary syndrome population where diabetes is both highly prevalent and a major driver of residual inflammatory risk. Moreover, imaging focused on the aorta and carotids rather than the coronary arteries, and although large-artery FDG uptake is a validated surrogate of vascular inflammation, the degree to which it tracks culprit-plaque biology and predicts coronary events in this specific setting remains to be seen. Finally, the absence of clinical events in the treatment group, while promising, must be interpreted cautiously given the small sample size and relatively short follow-up, and should primarily serve to motivate evaluation in a larger phase 3 outcomes trial.

Atherosclerotic plaque formation is driven by the complex interplay between lipid deposition, inflammatory changes, cell migration, and arterial wall injury. While statins remain foundational, and newer lipid-lowering strategies, such as PCSK9 inhibition and targeting lipoprotein(a) have further reduced atherogenic burden, a substantial residual cardiovascular risk persists, refocusing attention on inflammation as a therapeutic target. Large clinical trials have validated the principle that targeting inflammation can reduce cardiovascular events in selected populations,^3^ but they have also underscored that inflammation is not a single entity. Rather, it represents a broad and heterogeneous biological endotype governed by complex and sometimes opposing gene programs: some drive inflammatory escalation, while others coordinate its resolution. To date, most successful cardiovascular anti-inflammatory strategies have focused on components of innate immunity, including inhibition of IL-1β, IL-6 signaling, and the NLRP3 inflammasome. Now, the IVORY trial introduces a complementary paradigm: selectively augmenting endogenous adaptive immunity regulation through cytokine supplementation, can act upstream of innate inflammatory pathways and potentially complement or provide an alternative to direct cytokine inhibition.

A parallel advance with this trial is the emergence of inflammation imaging as a pharmacodynamic and stratification tool. Beyond PET, coronary CT angiography derived measures of perivascular adipose tissue can capture the arterial wall’s inflammatory “footprint”.^4^ Recent studies on machine-learning approaches link perivascular radiomic patterns to arterial transcriptomic changes and enable radiotranscriptomic signatures that represent scalable “virtual biopsies” of vascular inflammation that have the potential to identify patients with a specific immune endotype.^5^

In conclusion, immune regulation with low-dose IL-2 can modulate arterial inflammation after acute coronary syndromes, and it strengthens the case for larger trial validation. These developments point toward a future of precision immuno-cardiology in which inflammation is not just measured, but actively steered, making targeted inflammation therapy one of the most promising next frontiers in cardiology.

**Fig. 1:**
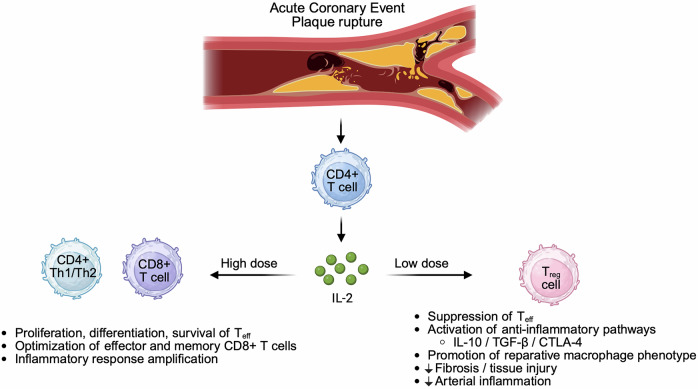
Schematic illustrating how interleukin-2 (IL-2) can exert contrasting immunological effects after acute coronary syndromes/myocardial infarction (ACS/MI) in a dose-dependent manner. Plaque rupture triggers antigen-driven T-cell activation, with activated CD4⁺ T cells producing IL-2. At high doses, IL-2 preferentially supports the proliferation, differentiation and survival of effector T cells (*T*_eff_), including CD4⁺ Th1/Th2 and CD8⁺ cytotoxic T cells, and promotes generation of effector and memory CD8⁺ responses, thereby amplifying inflammatory programmes. At low doses, IL-2 preferentially expands regulatory T cells (*T*_reg_), which express the high-affinity IL-2 receptor (CD25) and require IL-2 signaling to maintain FOXP3 expression and suppressive function. Expanded T_reg_ cells attenuate *T*_eff_ activity through contact-dependent and soluble mediator mechanisms, engage anti-inflammatory pathways (including IL-10, TGF-β and CTLA-4 signaling), promote reparative macrophage phenotypes and limit fibrosis, tissue injury, and inflammation. This illustration was created with BioRender

## Supplementary information

Author Checklist

## References

[1] Sriranjan-Rothwell, R. S. et al. Anti-inflammatory therapy with low-dose IL-2 in acute coronary syndromes: a randomized phase 2 trial. *Nat. Med*. 1–9 (2026). 10.1038/s41591-025-04090-y PMC12920103 41507574

[2] Wang, L. et al. Regulatory T cells in homeostasis and disease: molecular mechanisms and therapeutic potential. *Signal Transduct Target Ther.* **10**, 345 (2025). 41087343 10.1038/s41392-025-02326-4 PMC12521743

[3] Döring, Y., van der Vorst, E. P. C. & Weber, C. Targeting immune cell recruitment in atherosclerosis. *Nat. Rev. Cardiol.* **21**, 824–840 (2024). 38664575 10.1038/s41569-024-01023-z

[4] Chan, K. et al. Inflammatory risk and cardiovascular events in patients without obstructive coronary artery disease: the ORFAN multicentre, longitudinal cohort study. *Lancet* **403**, 2606–2618 (2024). 38823406 10.1016/S0140-6736(24)00596-8 PMC11664027

[5] Kotanidis, C. P. et al. Constructing custom-made radiotranscriptomic signatures of vascular inflammation from routine CT angiograms: a prospective outcomes validation study in COVID-19. *Lancet Digit Health* **4**, e705–e716 (2022). 36038496 10.1016/S2589-7500(22)00132-7 PMC9417284

