# Supplementary material for: Arterial inflammation after myocardial infarction: regulating the immune system
Source: Signal Transduct Target Ther. 2026 Mar 24;11:110. doi: 10.1038/s41392-026-02628-1 (PMC13018561; doi:10.1038/s41392-026-02628-1)
Supplement: Supplementary file 1 — Author Checklist [file 41392_2026_2628_MOESM1_ESM.pdf]

Author Checklist for Research Highlight

Corresponding author(s): Christos P. Kotanidis

Last updated by author(s): 27 January 2026

- [x] **Words** (1,100 words max excluding references and figure legend).
- [x] The **title** is suggested to be in no more than **15** words.
- [x] The **title** should avoid use of jargon, abbreviations, and question; **if abbreviation is indispensable, please put full name first and abbreviation in the brackets**.
- [x] **Authorship** (no more than 3 authors).
- [x] Please **double check** the authorship (including but not limited to the **spellings of all authors' names**, the **order of the author list**, and the **markings of all co-first and co-corresponding authors**) in both the manuscript files uploaded and the online submission system, to **ensure no mistakes**.
- [x] **Any changes to authorship will not be allowed once the manuscript has been accepted for publication. Correction Note regarding authorship changes will not be allowed either after formal publication.
- [ ] Please upload the "**Change of Authorship Request Form**" when submitting the revision if any changes concerning the authorship (including but not limited to: **removal** and/or **addition** of any authors, **change of authors' order**, etc.) are made. You can download this form at: https://resource-cms.springernature.com/springer-cms/rest/v1/content/7454878/data/v5
- [x] **Author institutions or affiliations** (as concise as possible).
- [x] **DO NOT** put the first one or two sentences (the brief introduction) into bold font.
- [x] **Figure**: Max of 1; should be uploaded as a single JPG or TIFF file.
- [x] **Image** resolution of **at least 300 dpi** at publication size.
- [x] **Figures** divided into parts should be labelled with a **lower-case, boldface** 'a', 'b', etc.in the **top left-hand** corner.
- [x] **Figures** use the same typeface Arial for all figures. Use symbol font for Greek letters.
- [x] **Figures** are best prepared at the size you would expect them to appear in print. At this size, the **optimum font size is 8pt** and no lines should be thinner than 0.25pt (0.09 mm).
- [x] **Figures** (or parts of figures) generated from online tools (e.g., **BioRender, etc**) or including online picture resources (e.g. from **SMART - Servier Medical ART, etc**) should be clearly indicated by **citing the original websites in the figure legend** or following the requirements mentioned in the original websites.
- [x] **Reference style** (All authors should be listed for papers with up to 5 authors; for papers with more than 5 authors, the first only should be listed, followed by et al. Abbreviations for titles of medical periodicals should conform to those used in the latest edition of Index Medicus. The first and last page numbers for each reference should be provided. References to the literature in the text should be typed as **superscripts after any punctuation**.)
- [x] **Reference number**: Max of 5.

☐ When describing COVID-19-associated background and results, please **avoid using** the words such as “Wuhan virus/strain”, “Hubei”, “spread from China”, etc.

☒ “**Conflicts of interest**” and “**Author contributions**” sections are **necessary**. Please add the statement “All authors have read and approved the article” in the Contributions part.

☒ **World map** is strongly **discouraged** from being used in figures.

☒ **Caution: please carefully check the figure or table to ensure no mistakes (spelling, grammar and common sense, etc.), misinterpretations and inconsistency with the paper highlighted. Such problems cannot be solved by publishing a Correction note after formal publication. Please pay attention to this issue.**

☒ **Figure style** (Please refer to Example 1 and 2)

Example 1:

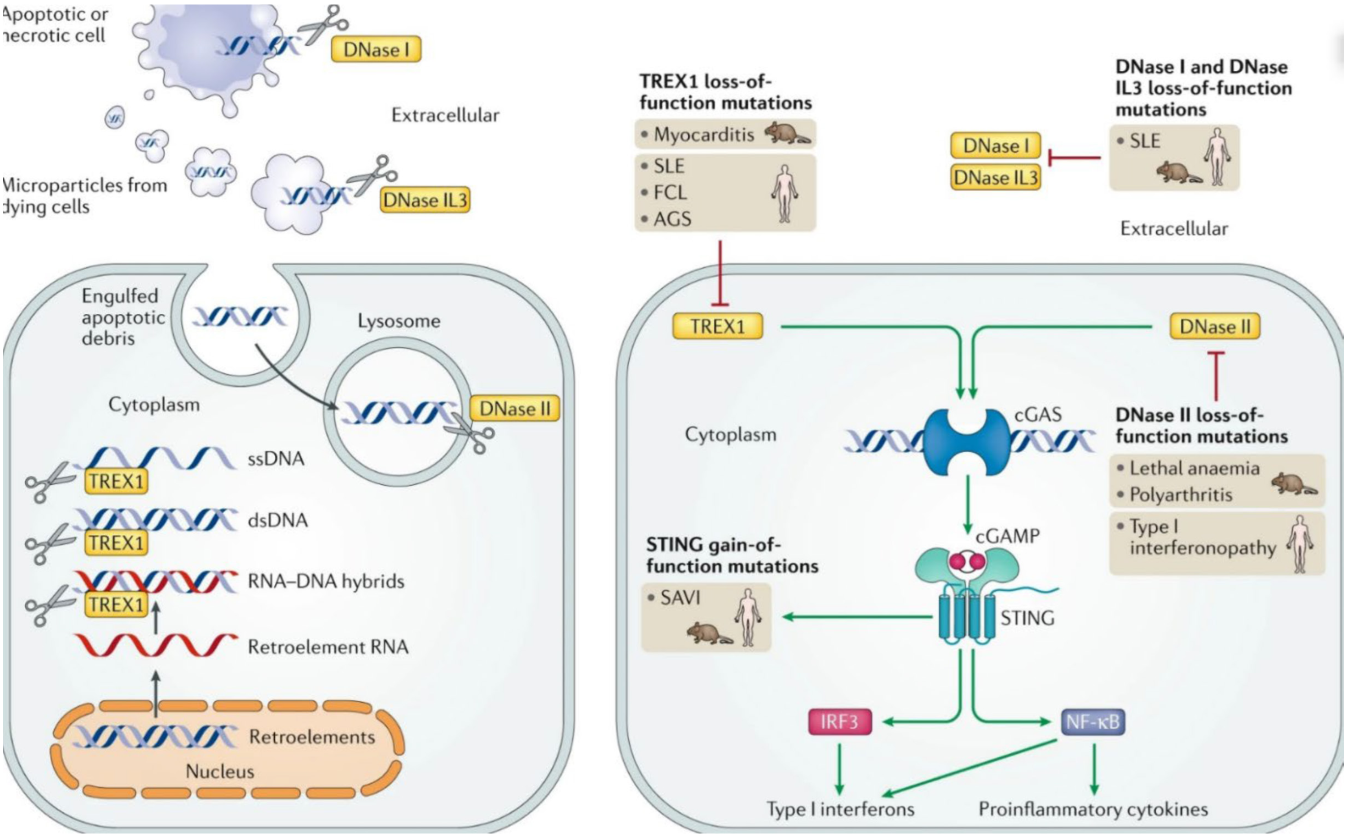

Example 2:

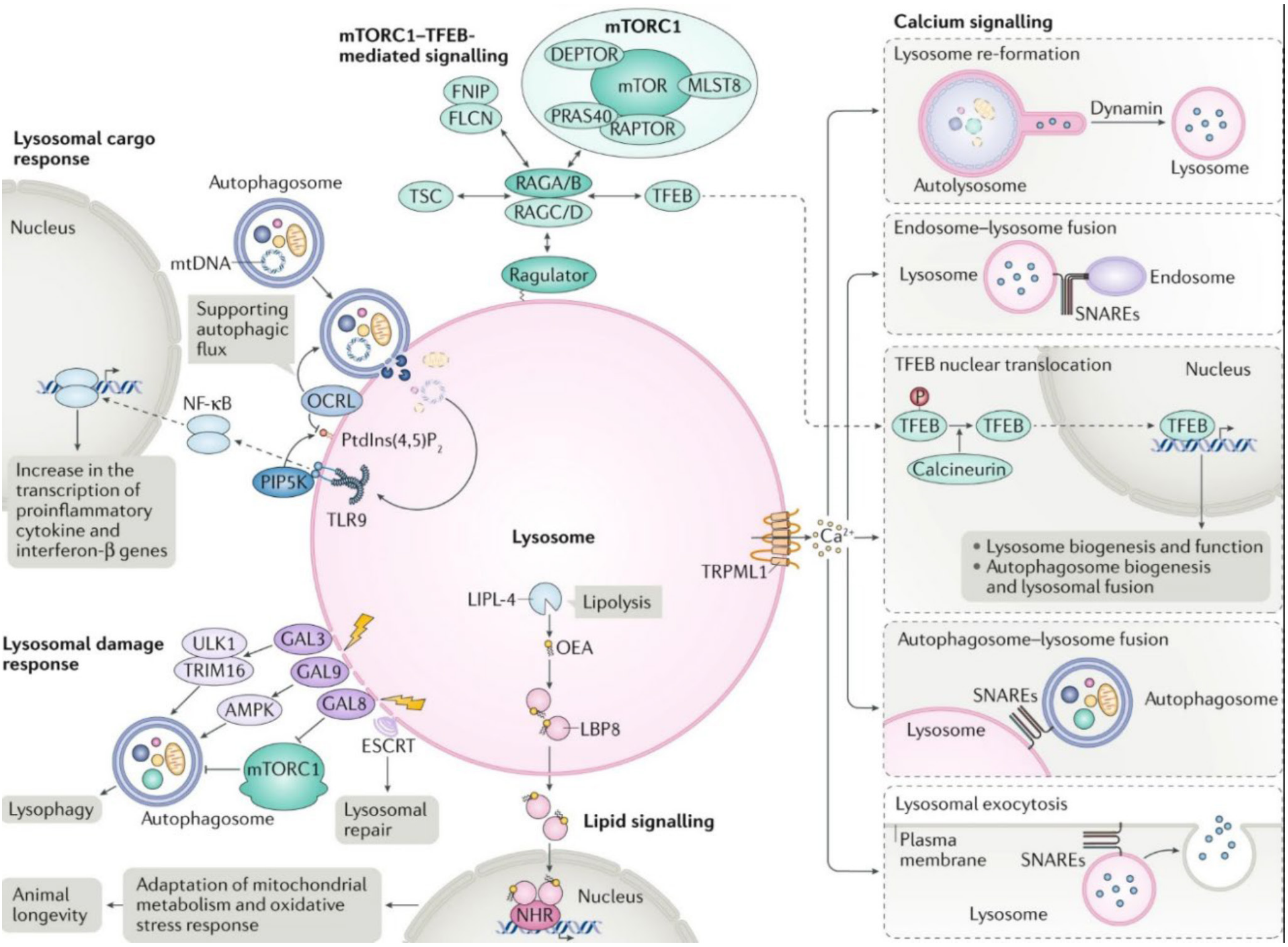

mTORC1–TFEB-mediated signalling
mTORC1
DEPTOR
mTOR
MLST8
PRAS40
RAPTOR
FNIP
FLCN
TSC
RAGA/B
RAGC/D
TFEB
Ragulator
Lysosomal cargo response
Nucleus
Autophagosome
mtDNA
Supporting autophagic flux
OCRL
NF-κB
PtdIns(4,5)P2
PIP5K
TLR9
Increase in the transcription of proinflammatory cytokine and interferon-β genes
Lysosome
TRPML1
Ca2+
LIPL-4
Lipolysis
OEA
LBP8
Lysosomal damage response
ULK1
TRIM16
GAL3
GAL9
GAL8
AMPK
ESCRT
mTORC1
Lysophagy
Autophagosome
Lysosomal repair
Lipid signalling
Nucleus
NHR
Animal longevity
Adaptation of mitochondrial metabolism and oxidative stress response
Calcium signalling
Lysosome re-formation
Dynamin
Autolysosome
Lysosome
Endosome–lysosome fusion
Lysosome
Endosome
SNAREs
TFEB nuclear translocation
Nucleus
P
TFEB
TFEB
TFEB
Calcineurin
• Lysosome biogenesis and function
• Autophagosome biogenesis and lysosomal fusion
Autophagosome–lysosome fusion
SNAREs
Autophagosome
Lysosome
Lysosomal exocytosis
Plasma membrane
SNAREs
Lysosome
